# TESTING PSYCHOLOGICAL RESILIENCE IN THE OLDER US POPULATION

**DOI:** 10.1093/geroni/igae098.1679

**Published:** 2024-12-31

**Authors:** Miles Taylor, Dawn Carr

**Affiliations:** Florida State University, Tallahassee, Florida, United States; Florida State University, Tallahassee, Florida, United States

## Abstract

The concept of resilience, broadly defined as the ability to successfully navigate or bounce back after stressors or traumas, has gained increased attention in several disciplinary literatures on aging. Certain challenges and setbacks, such as chronic condition onset and widowhood, become more normative in older adulthood. It is increasingly recognized that resilience is important to understanding health processes and independent functioning in later life. Aging scholars note that resilience can be operationalized as a set of individual characteristics promoting adaptation, a process of adaptation and/or coping, and healthy or beneficial outcomes after stressful setbacks or challenges. We focus on psychological resilience (PR), an individual’s capacity to navigate adversity through positive adaptation in a manner that protects health and well-being. Although numerous measures of PR exist, most are not incorporated into large longitudinal data on older adults that may provide insights on how this resource works across a variety of stressors and outcomes. In this session, we first discuss our collective work that has 1. innovated the creation of a PR measure that can be used in the Health and Retirement Study (HRS), and 2. tested the validity and predictive efficacy of this measure in this nationally representative data source. We then present key findings from our research on the protective effects of PR across a number of stressors and outcomes, showing how its connection to health processes may be helpful in shaping future interventions.

